# Recurrence of Autoimmune Hepatitis After COVID-19 Vaccination

**DOI:** 10.7759/cureus.25339

**Published:** 2022-05-25

**Authors:** Jon Elliott D Brubaker, Christopher L Casaccio, Michael J Brazeau

**Affiliations:** 1 Internal Medicine, Wright State University Boonshoft School of Medicine, Dayton, USA; 2 Internal Medicine, Wright-Patterson Air Force Base (AFB) Medical Center, Dayton, USA; 3 Gastroenterology, Wright-Patterson Air Force Base (AFB) Medical Center, Dayton, USA

**Keywords:** mrna-based vaccine, adverse reaction from vaccination, covid-19 vaccination, covid-19, autoimmune hepatitis

## Abstract

A 35-year-old Asian female with a pertinent past medical history of autoimmune hepatitis presented with an acute recurrence of autoimmune hepatitis two weeks after receiving the second dose of the Pfizer-BioNTech messenger RNA (mRNA) coronavirus disease 2019 (COVID-19) vaccine. Nine cases of autoimmune hepatitis after the administration of the COVID-19 vaccine have been reported, but this is the first documented case of a reactivation of autoimmune hepatitis in remission. As recommendations for COVID-19 vaccinations and booster shots continue to evolve, all adverse events should be reported to better educate and monitor patients.

## Introduction

The coronavirus disease 2019 (COVID-19) pandemic has ushered in the first use of messenger RNA (mRNA) vaccines. On December 11, 2020, the Food and Drug Administration granted emergency use authorization (EUA) to the Pfizer-BioNTech mRNA COVID-19 vaccine. Subsequently, EUA was granted to the Moderna COVID-19 vaccine on December 18, 2020. Messenger RNA directs cells to build foreign proteins leading to the desired immunologic response. It is a versatile technology that will likely continue to be a very prominent part of vaccine development in the future. After millions of mRNA vaccines had been administered, the Centers for Disease Control and Prevention granted full approval to the Pfizer-BioNTech vaccine on August 23, 2021. As must be expected with all medical interventions, cases of adverse events from the vaccine are slowly being reported. Prior to this publication, there were nine cases reported of autoimmune hepatitis that developed after the administration of the COVID-19 vaccine [1-9].

Autoimmune hepatitis is characterized by a cell-mediated attack against liver cells resulting in chronic hepatocellular necrosis, inflammation, and fibrosis, which can result in cirrhosis and liver failure. It is typically responsive to glucocorticoid and immunosuppressive therapy. The onset of autoimmune hepatitis can be abrupt or insidious, and recurrent attacks are not uncommon. The 10-year survival for autoimmune hepatitis is 80-98% with treatment and approximately 67% without treatment [10].

## Case presentation

A 35-year-old Asian female was originally diagnosed with autoimmune hepatitis in 2016 after her liver enzymes were noted to be elevated on routine labs: aspartate transferase (AST): 255 U/L, alanine transferase (ALT): 433 U/L, alkaline phosphatase: 74 U/L, and total bilirubin: 0.82 mg/dL. Her only past medical history included chronic sinusitis and insomnia. She had no family history of liver disease. Hepatitis A, B, and C viruses were negative. Cytomegalovirus was negative. Ferritin was elevated at 256 ng/ml. Smooth muscle antibody was elevated at 1:640 with IgG at 2382 mg/dL and antinuclear antibody (ANA) at 1:640 in a homogenous pattern. Liver biopsy was consistent with grade 2 stage II chronic hepatitis with lymphocytic infiltrate, piecemeal necrosis, and interface hepatitis. The patient was started on prednisone 60 mg followed by a taper and azathioprine 50 mg. Her liver enzymes normalized after treatment and she stopped taking azathioprine in November 2018. Her only complaint when treatment was discontinued was intermittent epigastric pain that had occurred for years. In December 2020, her AST level was 28 U/L, ALT was 29 U/L, and smooth muscle, mitochondrial, and parietal cell antibodies were all negative, indicating continued remission off therapy.

The patient received her first dose of the Pfizer-BioNTech mRNA COVID-19 vaccine on May 20, 2021, and her second dose on June 10, 2021. Two weeks after her second dose, her liver enzymes were elevated for the first time since her initial diagnosis in 2016; her AST was 129.5 U/L, ALT was 217 U/L, alkaline phosphatase was 72 U/L, and total bilirubin was 0.9 mg/dL. Smooth muscle antibody was positive at 1:20 with negative mitochondrial antibody and parietal cell antibody. She denied any abdominal pain, nausea, vomiting, weight loss, fevers, chills, malaise, fatigue, decreased appetite, or changes in bowel movements. There were no other changes in her medical history, and she had not started any new medications. Fibroscan in June of 2021 (one week prior to the elevation of liver enzymes) was F0-F1, showing no steatosis. These findings were consistent with the recurrence of her autoimmune hepatitis. She was monitored off treatment and repeat liver enzymes two weeks later showed improvement. Three months later in September 2021, her liver enzymes normalized near her previous baseline (AST: 31 U/L; ALT: 34 U/L).

## Discussion

A patient with autoimmune hepatitis in remission and off of treatment presented with recurrence of autoimmune hepatitis with elevated liver enzymes and positive smooth muscle antibodies. She had previously been treated with prednisone and azathioprine but had been off of treatment since 2018. The only change in her medical management prior to the recurrence was the administration of the Pfizer-BioNTech mRNA COVID-19 vaccine. Nine previous cases of new-onset autoimmune hepatitis associated with COVID-19 vaccines have been reported, but our patient is the first case of recurrence of autoimmune hepatitis that was previously in remission.

There is debate within the medical literature on whether these associations are due to causality or coincidence. Whether the occurrence/recurrence in these 10 cases was due to the COVID-19 spike protein contained in the vaccine, the mechanism of mRNA vaccines, or simply a flare of autoimmune hepatitis unrelated to the vaccine is unclear. Multiple pathways of autoimmune dysregulation following COVID-19 vaccinations have been proposed, including cross-reactivity of spike protein and autoimmune liver cells and mRNA-specific pathways including RNA activation of the type I interferon pathway, but findings are inconclusive [5]. The first mechanism involves molecular mimicry and the idea that molecular similarities between the viral proteins (such as the spike protein used in the vaccine) and proteins in human tissue are similar enough in structure that the immune system is activated against both. There is thought that some of the lung damage seen in severe COVID-19 infections is due to this immune response [11]. Another possible way that the vaccines may be inducing an autoimmune response has to do with the inherent nature of mRNA and the immunogenicity of nucleic acids. The primary goal of the mRNA in these vaccines is to be translated into immunogenic proteins that the body will respond to. But prior to translation into these proteins, the mRNA is able to bind to pattern recognition receptors (PRRs) in the cell, which leads to the recognition of the mRNA by Toll-like receptors, retinoic acid-derived gene-I (RIG-I), melanoma differentiation-associated protein 5 (MDA5), and other proteins leading to activation of inflammatory cascades [12]. The activation of the type 1 interferon pathway and nuclear factor kappa B pathway, among others, may then lead to autoimmune responses seen in these cases. Overall, these 10 cases raise the possibility of autoimmune dysregulation caused by the COVID-19 vaccine.

## Conclusions

The COVID-19 pandemic has ushered in the era of mRNA vaccines. These vaccines, along with COVID-19 itself, have the potential to cause autoimmune dysregulation. While it is unclear if the vaccine caused the autoimmune dysregulation seen in our patient and others, the possibility exists. As COVID-19 policies continue to change and new vaccination guidelines are implemented, possible complications of vaccination should be part of our education and monitoring of patients. Further research is needed to investigate the possibility of autoimmune dysregulation with mRNA vaccines.
